# Multidisciplinary transcatheter rescue of post-infarction ventricular septal rupture in cardiogenic shock: expanding the role of percutaneous closure—case report

**DOI:** 10.1093/ehjcr/ytaf656

**Published:** 2025-12-16

**Authors:** Italo Menezes Ferreira, Raphael Paris Rosan, Rodrigo Dantas Ferraz, Bruno Querido Marcondes Santos, Carlos Augusto Cardoso Pedra, Antonio Tito Paladino Filho, Fausto Feres, Louis Nakayama Ohe

**Affiliations:** Cardiac Intensive Care Unit, Dante Pazzanese Institute of Cardiology, Avenida Doutor Dante Pazzanese 500, São Paulo, 04012180, Brazil; Cardiac Intensive Care Unit, Dante Pazzanese Institute of Cardiology, Avenida Doutor Dante Pazzanese 500, São Paulo, 04012180, Brazil; Cardiology Residency Program, Dante Pazzanese Institute of Cardiology, Avenida Doutor Dante Pazzanese 500, São Paulo, 04012180, Brazil; Cardiology Residency Program, Dante Pazzanese Institute of Cardiology, Avenida Doutor Dante Pazzanese 500, São Paulo, 04012180, Brazil; Department of Interventional Cardiology, Dante Pazzanese Institute of Cardiology, Avenida Doutor Dante Pazzanese 500, São Paulo, 04012180, Brazil; Department of Cardiovascular Imaging, Dante Pazzanese Institute of Cardiology, Avenida Doutor Dante Pazzanese 500, São Paulo, 04012180, Brazil; Department of Interventional Cardiology, Dante Pazzanese Institute of Cardiology, Avenida Doutor Dante Pazzanese 500, São Paulo, 04012180, Brazil; Department of Interventional Cardiology, Dante Pazzanese Institute of Cardiology, Avenida Doutor Dante Pazzanese 500, São Paulo, 04012180, Brazil

**Keywords:** Case report, Ventricular septal rupture, Myocardial infarction, Heart Team, Cardiogenic shock, Intra-aortic balloon pump, Percutaneous closure

## Abstract

**Background:**

Post-infarction ventricular septal rupture is a rare but often fatal mechanical complication of acute myocardial infarction, particularly when associated with cardiogenic shock and multiorgan dysfunction.

**Case summary:**

A 65-year-old woman presented in cardiogenic shock five days after initial chest pain. ECG showed ST-segment elevation in anterior and inferior leads. Coronary angiography revealed thrombotic occlusion of the proximal left anterior descending artery, successfully treated with primary percutaneous coronary intervention. Transthoracic echocardiography later revealed an 11 mm inferobasal ventricular septal rupture. Due to high surgical risk (EuroSCORE II >50%) in the setting of renal failure and recent dual antiplatelet therapy, the multidisciplinary Heart Team opted for delayed percutaneous closure. Fifteen days after symptom onset, a 16 mm CERA® occluder was successfully deployed with only trivial residual shunt. The patient showed rapid clinical improvement, was weaned from intra-aortic balloon pump and vasopressors, and discharged on Day 20 to cardiac rehabilitation.

**Discussion:**

This case illustrates the feasibility and efficacy of delayed percutaneous closure of post-infarction ventricular septal rupture in a critically ill patient. While surgery remains standard therapy, percutaneous closure offers a viable life-saving alternative in select high-risk patients, especially when delayed to allow scar formation. Mechanical circulatory support may serve as a bridge to intervention. Multidisciplinary Heart Team collaboration is essential for optimal individualized management.

Learning points

- Percutaneous treatment is a safe option in patients with post-MI VSR
- Pre-procedural intra-aortic balloon use is key to stabilizing the patient

## Introduction

Post-infarction ventricular septal defect (VSD) is a rare but catastrophic mechanical complication of acute myocardial infarction (AMI). While its incidence has declined to 0.2% in the reperfusion era, its prognosis remains grim. Inferior infarctions account for up to half of VSD cases, and surgical repair carries the highest mortality among cardiac procedures, especially when performed within the first week.^1^ Early diagnosis is often delayed by the overlap of clinical signs with other mechanical complications like papillary muscle rupture.

Management is complex and time sensitive. While surgery remains the gold standard, high operative mortality has prompted the exploration of less invasive alternatives. Recent advances in mechanical circulatory support (MCS) and device closure techniques have enabled percutaneous VSD closure as a salvage strategy in select patients.^2^ We present a case of successful transcatheter closure of an inferobasal VSD following extensive anterior STEMI and cardiogenic shock, managed through a multidisciplinary Heart Team approach.

## Summary figure

**Figure:**
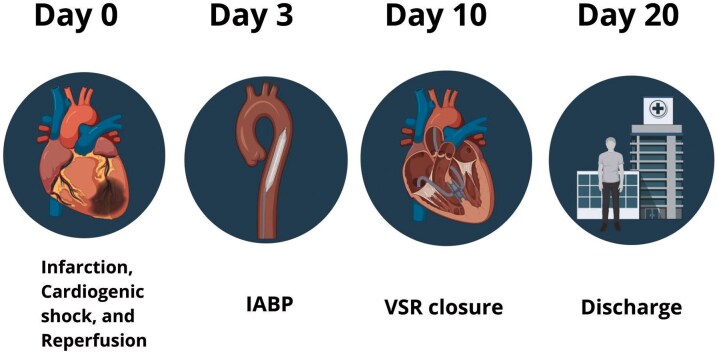


## Case presentation

A 65-year-old female with a history of hypertension, diabetes mellitus, and dyslipidaemia was referred to our tertiary care centre after 12 h of persistent chest pain and anterior ST-segment elevation. No fibrinolytic therapy had been administered despite the absence of contraindications. She reported a prodromal episode of chest discomfort 5 days prior, which progressively worsened, culminating in severe dyspnoea on the day of admission.

On arrival, the patient appeared tachypnoeic (28 breaths/min) and hypoxaemic (SpO₂ 93% on a non-rebreather mask at 10 L/min) and was receiving vasoactive support with norepinephrine (0.1 µg/kg/min) and dobutamine (13 µg/kg/min). Her blood pressure was 80/50 mmHg, heart rate 118 bpm, and capillary refill time 3 s. Cardiac auscultation revealed regular rhythm without murmurs, and pulmonary examination demonstrated bilateral inspiratory crackles extending to the mid-lung fields.

Initial laboratory investigations revealed a mixed acid–base disorder (pH 7.40, pCO₂ 33 mmHg, pO₂ 92 mmHg, bicarbonate 20.4 mmol/L, lactate 2.0 mmol/L). The patient was anaemic (haemoglobin 9.7 g/dL) and had signs of renal dysfunction, with elevated creatinine (2.1 mg/dL) and urea (85 mg/dL). Cardiac biomarkers were markedly elevated: high-sensitivity troponin I measured 21 250 ng/L (reference <11 ng/L), and NT-proBNP was 28 600 pg/mL.

**Figure 1:**
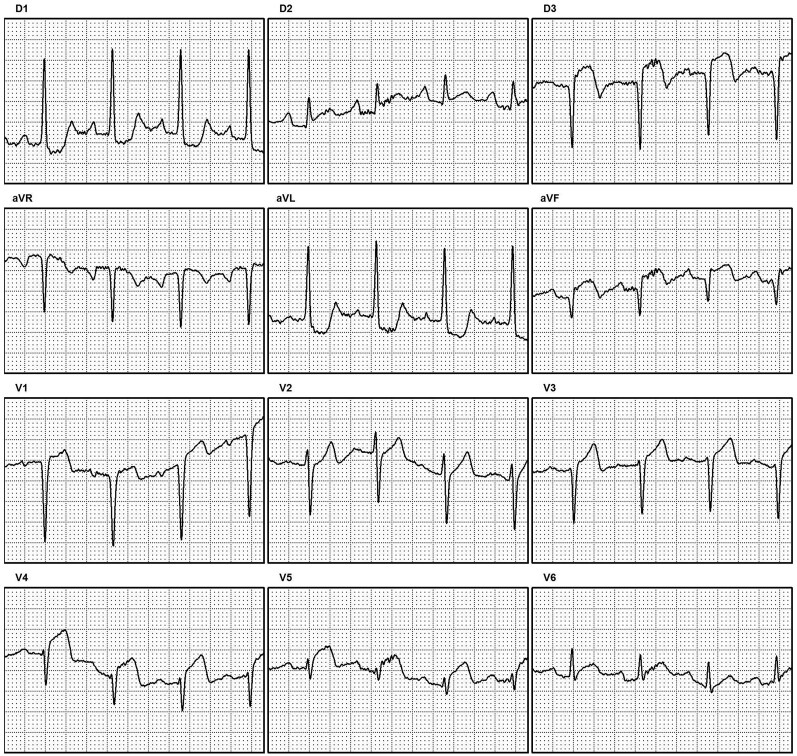
Admission 12-lead ECG demonstrating an inferior and anterior ST-elevation myocardial infarction (STEMI).

**Figure 2:**
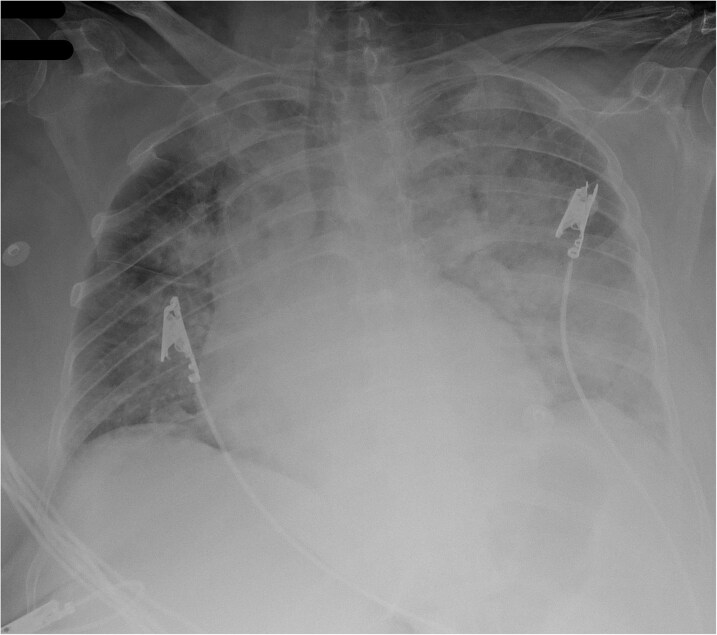
Admission chest X-ray revealed pulmonary congestion.

Electrocardiogram confirmed extensive anterior ST-elevation myocardial infarction (STEMI) with inferior involvement (*Figure 1*) and chest X-ray revealed pulmonary congestion (*Figure 2*). Based on these findings, the patient was classified as having SCAI Stage D cardiogenic shock.

Due to persistent hypotension and presumed ongoing infarction, the patient was promptly taken to the catheterization laboratory, in accordance with institutional protocol for cardiogenic shock. She received intravenous furosemide (100 mg) and underwent cautious titration of vasoactive agents, aiming to maintain adequate perfusion while minimizing oxygen supply–demand mismatch.

**Figure 3:**
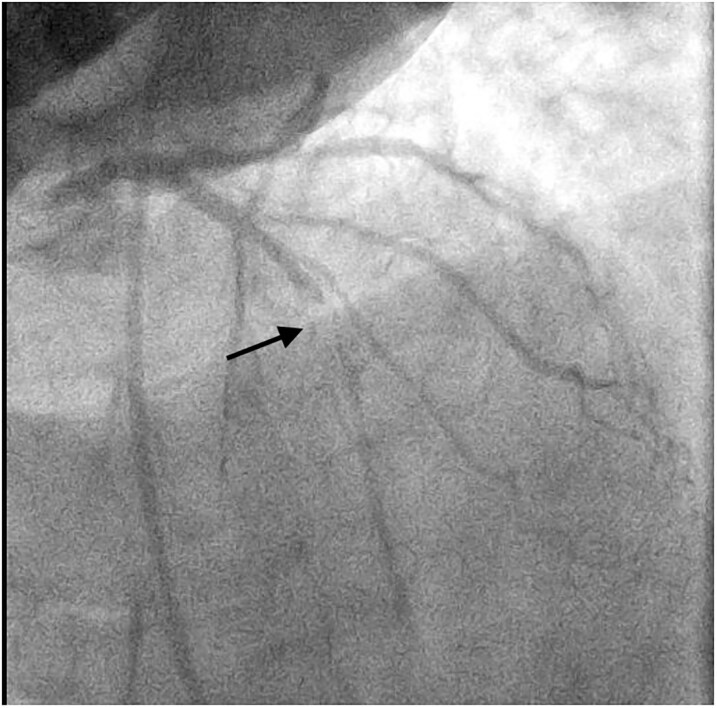
Initial coronary angiogram of the left anterior descending artery in the right cranial oblique projection.

**Figure 4:**
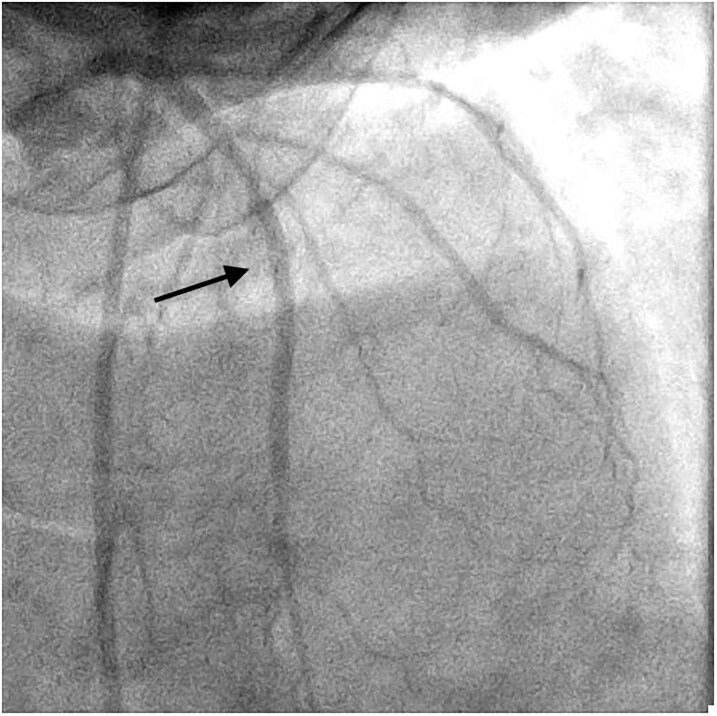
Post-angioplasty of the anterior descending artery.

Coronary angiography revealed a critical stenosis in the mid-left anterior descending (LAD) artery (*Figure 3*), which was successfully treated with primary PCI and TIMI 3 flow restoration (*Figure 4*). Ventriculography and right heart catheterization were deferred due to haemodynamic instability.

In the coronary care unit (CCU), norepinephrine was discontinued within 24 h, dobutamine was weaned to 8 µg/kg/min, and supplemental oxygen was reduced to 1 L/min via nasal cannula. However, on hospital day 3, the patient developed worsening dyspnoea and increased oxygen requirement.

Transthoracic echocardiography revealed a ventricular septal defect (VSD) located in the inferobasal portion of the interventricular septum. An intra-aortic balloon pump (IABP) was promptly inserted, resulting in rapid haemodynamic stabilization. Following IABP initiation, the patient remained stable, and dobutamine was further reduced to 5 µg/kg/min while maintaining adequate perfusion and oxygenation with only 1 L/min nasal oxygen. This dose was maintained until the definitive intervention. There was no further need for escalation of inotropic or ventilatory support. Despite the initial stabilization, the patient subsequently developed oliguric acute kidney injury (peak creatinine 5.4 mg/dL), necessitating continuous renal replacement therapy (CRRT).

**Figure 5:**
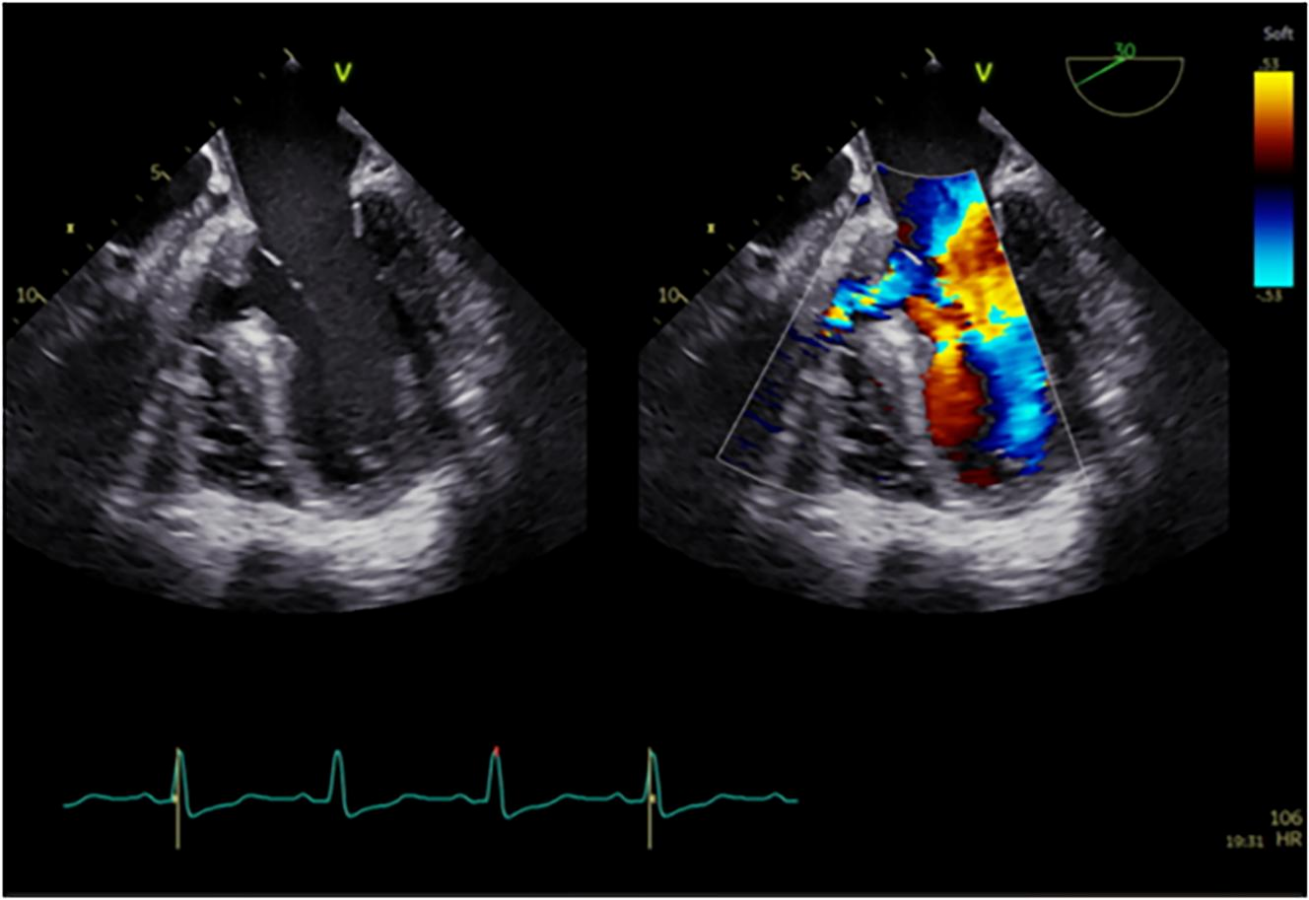
Transoesophageal echocardiography (TEE) revealed a ventricular septal rupture (VSR), measuring approximately 11 mm in diameter, with left-to-right shunting on colour Doppler.

Over the following 2 days, transoesophageal echocardiography (TEE) (*Figure 5*), complemented by three-dimensional imaging (3D-TEE), was performed, confirming an 11 mm left-to-right shunt with a left-to-right ventricular gradient of 25 mmHg, a preserved left ventricular ejection fraction of 60%, and an estimated pulmonary artery systolic pressure of 59 mmHg (see Supplementary material online, *Video 1*). Biventricular function was preserved, with no pericardial effusion. Regional wall-motion analysis demonstrated akinesia of the basal interventricular septum, with preserved contractility in the remaining segments. The mitral valve showed mild-to-moderate regurgitation, with thin cusps and normal leaflet mobility. Importantly, 3D-TEE provided detailed anatomic information sufficient to confirm the feasibility of percutaneous closure of the VSD.

A multidisciplinary Heart Team discussed all the management options. Surgical repair was deemed prohibitive (EuroSCORE II >50%) in view of cardiogenic shock, ongoing continuous renal replacement therapy (CRRT), and recent dual antiplatelet therapy (DAPT). Consequently, the decision was made to proceed with percutaneous closure.

On Day 10, the patient underwent transcatheter VSD closure under general anaesthesia and TEE guided. She was extubated 24 h later, with prompt haemodynamic and respiratory recovery.

**Figure 6:**
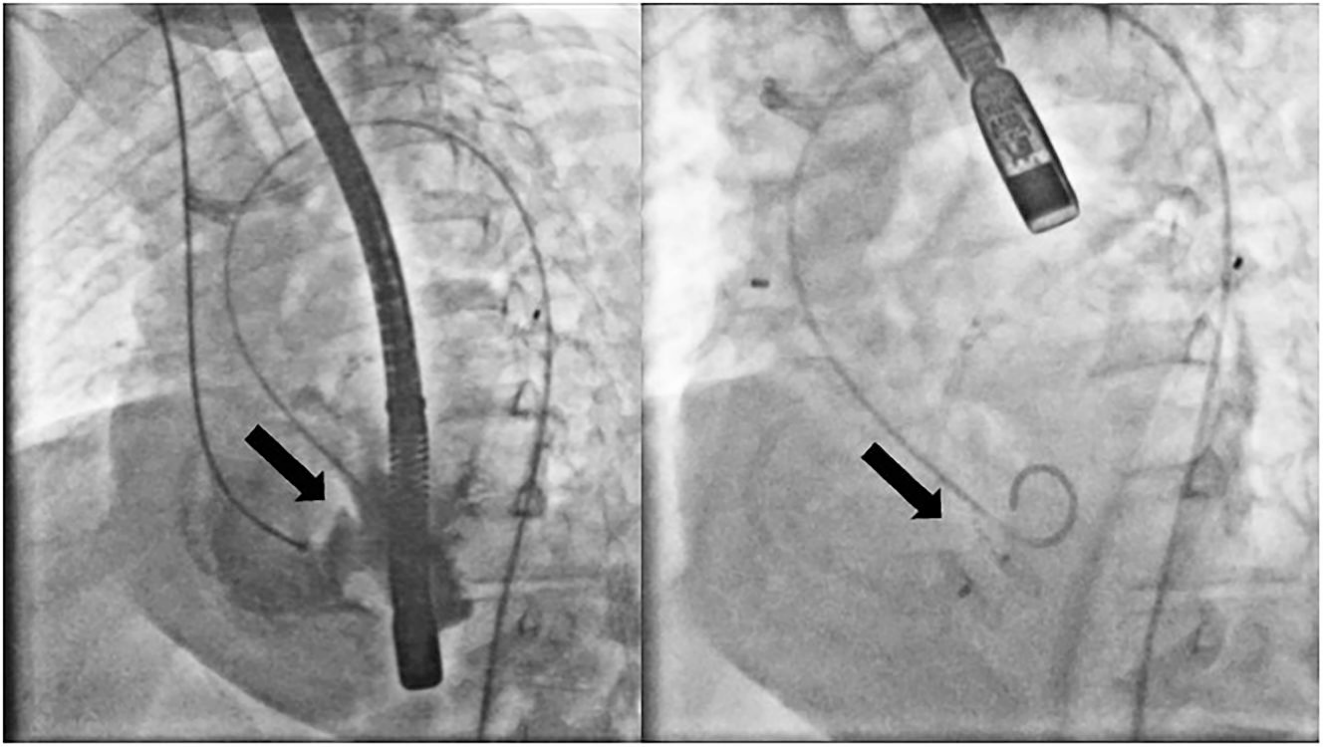
Angiogram of ventricular septal defect (left) and percutaneous closure of the ventricular septal defect (right).

**Figure 7:**
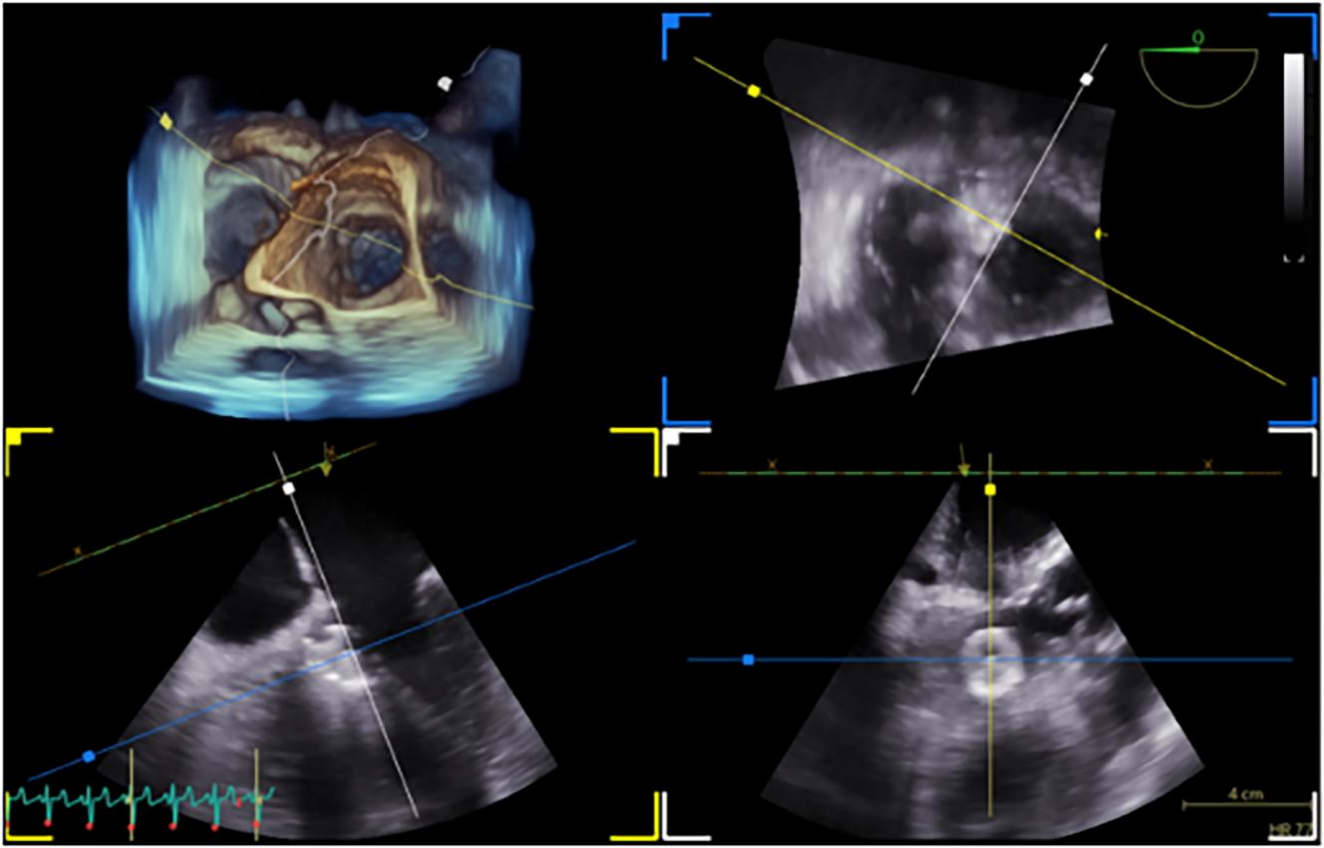
Intraprocedural transoesophageal echocardiogram was after the closure of the ventricular septal rupture.

A single arterial and single venous access approach was used, via the right femoral artery and the right internal jugular vein, respectively. A guidewire was advanced from the left to the right ventricle across the septal defect and retrieved through the venous route, creating an arteriovenous rail. A 16 mm CERA® muscular VSD occluder (50% oversizing) was then deployed. This dual-access strategy minimized vascular trauma and procedural complexity in this high-risk patient. After establishing the rail, the delivery sheath was advanced through the venous route, while the arterial catheter was exchanged for a pigtail catheter to facilitate control angiography (*Figure 6*, see Supplementary material online, *Videos 2* and *3*). Intraprocedural transoesophageal echocardiography (TEE) confirmed optimal device positioning with only a trivial residual shunt (*Figure 7*).

Following closure, the patient’s haemodynamics improved dramatically. Over the subsequent 10 days, inotropes were completely withdrawn, renal replacement therapy was discontinued, and supplemental oxygen was stopped. Importantly, systemic blood pressure increased steadily after elimination of the left-to-right shunt. This required the initiation and careful titration of intravenous nitroprusside, followed by the progressive reintroduction of her chronic antihypertensive regimen, which comprised six drug classes prior to admission. These changes clearly reflect both an improvement in cardiac output and the haemodynamic consequences of abolishing the shunt, corroborating the clinical impression of significant haemodynamic recovery after device closure.

She was discharged on hospital day 20 to a cardiac rehabilitation programme on guideline-directed medical therapy. At 1-month follow-up, she remained clinically stable.

## Discussion

This case highlights a rare but devastating mechanical complication of myocardial infarction: ventricular septal rupture (VSR). Despite modern reperfusion, VSR still carries high mortality, particularly when complicated by cardiogenic shock.^1,2^ Without closure—surgical or transcatheter—mortality can exceed 90% within weeks.^2^

VSR typically arises within 1 week post-transmural infarction, presenting with acute haemodynamic collapse. Risk factors include female sex, delayed presentation, and large infarcts^3^—all present here. In this case, mid-LAD occlusion caused extensive infarction with rupture in the inferobasal interventricular septum, as confirmed by TEE. Most anterior infarcts produce apical VSRs, but basal extension may occur. The surrounding necrotic myocardium is extremely friable in the acute phase, increasing the risk of procedural failure or device embolization if closure is attempted too early. Timely recognition of VSR is crucial but can be challenging.

The clinical presentation may overlap with other mechanical complications such as papillary muscle rupture or cardiac free-wall rupture. A new, harsh holosystolic murmur in a post-MI patient, particularly if radiating widely, mandates immediate imaging. Early diagnosis is essential but challenging. In our case, echocardiography was delayed until clinical deterioration on Day 3. According to the 2023 ESC Guidelines for Acute Coronary Syndromes, emergency transthoracic echocardiography is recommended in patients with suspected ACS presenting with cardiogenic shock or possible mechanical complications (Class I, Level C).^4^

In retrospect, a focused bedside echocardiogram could have been performed during transfer to the catheterization laboratory, potentially facilitating earlier recognition of a developing septal rupture. Had the ventricular septal rupture been identified at admission, a surgical repair combined with coronary revascularization might have been considered as the definitive treatment option, in accordance with current guideline recommendations for early surgical management of mechanical complications following myocardial infarction.^2,4^ However, the clinical and haemodynamic findings at presentation suggested evolving shock primarily driven by ongoing ischaemia, while mechanical complication was not initially suspected based on physical examination. This highlights the real-world, time-sensitive decision-making required when balancing diagnostic thoroughness and rapid reperfusion in cardiogenic shock.

The mainstay of treatment for post-MI VSR is definitive closure—historically achieved via surgical repair. However, early surgical repair (within 7 days of MI) is associated with very high operative mortality due to the friable myocardial tissue and frequent multiorgan failure in this period.^5,6^

Delaying surgery allows time for infarct healing and scar formation, which strengthens the septal tissue and can dramatically reduce surgical risk. Indeed, multiple surgical series have documented operative mortality well above 50% when repair is attempted in the first week, vs. under 10% if intervention can be postponed beyond 3–4 weeks.^7^

Unfortunately, many patients, including ours, cannot tolerate a prolonged delay on conservative therapy alone, as ongoing shunting and heart failure rapidly lead to demise. Mechanical circulatory support (MCS) can stabilize unstable patients until a safer window for closure is reached. IABP was chosen to reduce afterload and left-to-right shunting and improve coronary perfusion, buying valuable time for tissue healing prior to closure. Although the use of intra-aortic balloon pump (IABP) in cardiogenic shock was not shown to improve mortality in the general AMI population in the IABP-SHOCK,^8^ it excluded mechanical complications. Thus, its findings should not be extrapolated to VSR. The 2023 ESC guidelines (Class IIa) and the latest ACC/AHA guidelines support IABP in this setting.^5,9,10^

ECMO was considered but not pursued: our institution lacks routine ECMO capacity, and the patient was already improving with IABP. Although ECMO has been used as a bridge to transplant or delayed repair,^11^ these strategies are centre-specific and patient-dependent. Transplantation was not considered as first-line in 65-year-olds with preserved LVEF and no irreversible damage. Furthermore, she was responding to support and had improving multiorgan function. In our patient, IABP support stabilized haemodynamics, enabled dialysis, and avoided further escalation of dobutamine or oxygen, effectively bridging to closure.

The Heart Team initially considered emergent surgery; however, the EuroSCORE II was >50%, and the concurrent need for inotropes and dialysis, together with recent dual antiplatelet therapy (DAPT), rendered the operative risk prohibitive. Notably, patients who proceed to surgery while continuing clopidogrel up to the day of operation have roughly double the risk of major mediastinal bleeding, a substantially higher likelihood of re-exploration for haemorrhage, and greater transfusion requirements—findings that are similar with prasugrel or ticagrelor.^12^

We used 3D transthoracic echocardiography for VSR sizing due to its real-time availability, acceptable resolution, and bedside feasibility. Although the spatial resolution of 3D-TEE is lower than that of cardiac magnetic resonance or cardiac computed tomography, it was our method of choice to precisely delineate the ventricular septal rupture, providing sufficient anatomical detail to characterize defect morphology and evaluate feasibility for percutaneous intervention while remaining readily applicable in the ICU setting owing to an excellent acoustic window.

For the percutaneous closure, we selected the CERA® VS occluder due to its double-disc nitinol design, which anchors well on either side of the septum once fibrotic tissue has formed. Standard practice is to oversize the device relative to the defect (we chose 16 mm for an ∼11 mm tear, ∼45% larger) to ensure secure seating and minimize residual shunt.^13^

Forming the arteriovenous rail (by snaring the guidewire in the RV and externalizing through an arterial sheath) provided a stable track to deliver the closure device across the septum. Transoesophageal echo guidance was invaluable throughout positioning and deployment, confirming the device’s proper placement and only trivial residual flow post-implantation. Percutaneous VSR closure, while less invasive than surgery, is not without risk. Reported complications include device embolization, significant residual shunts, ventricular arrhythmias, haemolysis, and even extension of the septal rupture due to device stress.^14^

Moreover, the overall mortality of transcatheter closure remains high given the critical condition of these patients. A systematic review reported successful device deployment in ∼89% of cases, but an in-hospital mortality of 32% even with intervention.^15^ In our case, fortunately, none of the major complications occurred. The device remained well-seated with no embolization and only a tiny residual shunt.

The patient improved rapidly after closure and was weaned off all support within a day, underscoring that percutaneous therapy can be a lifesaving option in carefully selected patients who are not surgical candidates.

Ultimately, this case emphasizes the importance of multidisciplinary decision-making in the management of post-infarction VSR, especially in a haemodynamically unstable patient. The integration of advanced imaging, interventional cardiology, cardiac surgery, and intensive care expertise allowed for a tailored, stepwise approach in our patient: from emergent PCI for coronary reperfusion, to temporary stabilization with IABP (and renal support), to a successful delayed transcatheter septal closure once the tissue was more amenable.

As clinical experience and device technology continue to advance, transcatheter closure is emerging as a viable alternative to surgery for post-MI VSR in patients who are too high risk for immediate surgery. While randomized trials are unlikely given the rarity and acuity of this complication, accumulating real-world cases and series support the growing role of percutaneous therapy as a lifesaving bridge or definitive treatment in this otherwise dire condition.

## Lead author biography


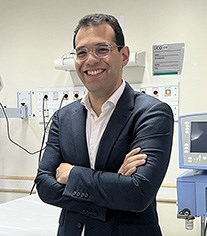


Dr. Italo Menezes Ferreira graduated from the Federal University of Piauí (UFPI) and completed his residency in Cardiology at the Dante Pazzanese Institute of Cardiology in São Paulo, Brazil, where he currently serves as Coordinator of the Cardiac Intensive Care Unit-Coronary Care Unit. His clinical and research interests include acute coronary syndromes, mechanical complications of myocardial infarction, and critical care cardiology. He is actively involved in multidisciplinary Heart Team decision-making. Dr. Ferreira is also pursuing a doctoral degree focused on high-sensitivity troponin I in populations with established coronary artery disease.

## Supplementary Material

ytaf656_Supplementary_Data

## Data Availability

All relevant data are included in the article. No additional data are available.
