# Supplementary material for: Multidisciplinary transcatheter rescue of post-infarction ventricular septal rupture in cardiogenic shock: expanding the role of percutaneous closure—case report
Source: Eur Heart J Case Rep. 2025 Dec 16;10(1):ytaf656. doi: 10.1093/ehjcr/ytaf656 (PMC12810202; doi:10.1093/ehjcr/ytaf656)
Supplement: ytaf656_Supplementary_Data [file ytaf656_supplementary_data.zip › Supplementary_Legends.docx]

## **Supplemental videos legends**

**Supplementary Video 1**: Transoesophageal echocardiography showing the inferobasal ventricular septal rupture with a left-to-right shunt prior to device closure.

**Supplementary Video 2**: Intraprocedural angiogram performed immediately before device implantation.

**Supplementary Video 3:** Post-procedural control angiogram confirming optimal device position and effective shunt elimination.
